# Anti-*Plasmodium falciparum* Activity of Extracts from 10 Cameroonian Medicinal Plants

**DOI:** 10.3390/medicines5040115

**Published:** 2018-10-29

**Authors:** Toghueo Kouipou Rufin Marie, Heroine Mbetyoumoun Mfouapon, Eugenie Aimée Madiesse Kemgne, Cedric Derick Jiatsa Mbouna, Patrick Valere Tsouh Fokou, Dinkar Sahal, Fabrice Fekam Boyom

**Affiliations:** 1 Antimicrobial & Biocontrol Agents Unit (AmBcAU), Laboratory for Phytobiochemistry and Medicinal Plants Studies, Department of Biochemistry, Faculty of Science, University of Yaoundé I, Yaoundé P.O. Box 812, Cameroon; toghueo.rufin@yahoo.fr (R.M.K.T.); mshermfouapon@yahoo.fr (H.M.M.); eugboyom@yahoo.fr (E.A.M.K.); cedrickjiatsa@yahoo.com (C.D.J.M.); Tsouh80@yahoo.fr (P.V.T.F.); 2 Malaria Research Laboratory, International Centre for Genetic Engineering and Biotechnology, New Delhi 110067, India

**Keywords:** *Plasmodium falciparum*, medicinal plants, antiplasmodial activity, selectivity index

## Abstract

**Background:** In the midst of transient victories by way of insecticides against mosquitoes or drugs against malaria, the most serious form of malaria, caused by *Plasmodium falciparum*, continues to be a major public health problem. The emergence of drug-resistant malaria parasites facilitated by fake medications or the use of single drugs has worsened the situation, thereby emphasizing the need for a continued search for potent, safe, and affordable new antimalarial treatments. In line with this need, we have investigated the antiplasmodial activity of 66 different extracts prepared from 10 different medicinal plants that are native to Cameroon. **Methods:** Extracts were evaluated for their capacity to inhibit the growth of the chloroquine-sensitive (*Pf*3D7) and resistant (*Pf*INDO) strains of *P. falciparum* using the SYBR green fluorescence method. The cytotoxicity of promising extracts against human embryonic kidney cells (HEK293T) mammalian cells was assessed by MTT assay. **Results:** The antiplasmodial activity (50% inhibitory concentration, IC_50_) of plant extracts ranged from 1.90 to >100 μg/mL against the two strains. Six extracts exhibited good activity against both *Pf*3D7 and *Pf*INDO strains, including cold water, water decoction, and ethyl acetate extracts of leaves of *Drypetes principum* (Müll.Arg.) Hutch. (IC_50_3D7/INDO = 4.91/6.64 μg/mL, 5.49/5.98 μg/mL, and 6.49/7.10 μg/mL respectively), water decoction extract of leaves of *Terminalia catappa* L. (IC_50_3D7/INDO = 6.41/8.10 μg/mL), and water decoction extracts of leaves and bark of *Terminalia mantaly* H.Perrier (IC_50_3D7/INDO = 2.49/1.90 μg/mL and 3.70/2.80 μg/mL respectively). These promising extracts showed no cytotoxicity against HEK293T up to 200 μg/mL, giving selectivity indices (SIs) in the range of >31.20–80.32. **Conclusions:** While providing credence to the use of *D. principum*, *T. catappa*, and *T. mantaly* in the traditional treatment of malaria, the results achieved set the stage for isolation and identification of active principles and ancillary molecules that may provide us with new drugs or drug combinations to fight against drug-resistant malaria.

## 1. Introduction

Malaria is one of the world’s most severe and deadly infectious diseases, and primarily affects the most disadvantaged populations. In fact, approximately 216 million cases of malaria and 445,000 attributed deaths were reported globally in 2016 [1]. Of these, about 91% of total deaths were in Africa, with pregnant women and children under 5 years being the most affected groups [2]. In Cameroon, which is among the most affected countries, 71% of the population lives in high-transmission areas [3]. Moreover, effective eradication strategies have been elusive, primarily owing to the complex life cycle of *Plasmodium* and the emergence of drug-resistant strains of *Plasmodium falciparum*, the most lethal *Plasmodium* species in humans [4,5,6]. Against this background and in the absence of any credible vaccine, there is urgent need to discover new, potent, safe, and affordable drugs to combat malaria.

The rich ethnopharmacological history of traditional knowledge and usage associated with medicinal plants represents a rich collection of bioactive substances as gifts of nature to mankind. The approach of retrieval of information from the folk use of plants has often yielded more potentially useful compounds than the empirical approach [7,8,9]. It is well known that in rural populations in Africa, Asia, and South America, people often use ethnobotanical and ethnomedical alternatives for the management of health problems including malaria. Such alternative remedies have provided leads for the development of drugs useful in therapeutics as practiced in Western medicine [10,11,12]. Indeed, some well-known examples of the seminal contributions of ethnomedicine to the treatment of malaria by the modern medicine way are quinine and artemisinin, isolated from *Cinchona* tree and *Artemisia annua*, respectively [13]. In this vein, the present paper reports the antiplasmodial potential of extracts from nine medicinal plants (*Alchornea lacifolia* Sw., *Annona senegalensis* Pers., *Cananga odorata* (Lam.) Hook.f. & Thomson, *Occimum gratissimum* L., *Senna alata* (L.) Roxb., *Terminalia catappa* L., *Terminalia mantaly* H.Perrier, *Ficus benjamina* L. and *Ficus exasperata* Vahl) traditionally used for the treatment of malaria or associated symptoms in Cameroon [14,15,16,17,18,19,20,21,22] and one (*Drypetes principum* (Müll.Arg.) Hutch.) with no previous reports.

Except for *D. principum*, the antiplasmodial activities of all other plants listed above have been reported previously [21,22,23,24,25,26,27]. However, all these studies have been with whole plants and none of these has assessed which plant part and extraction solvent are best suited for obtaining the highest antiplasmodial activity. In the present study we have prepared extracts of individual plant parts using different solvents and determined the antiplasmodial potency and selectivity of each. 

## 2. Materials and Methods

### 2.1. Collection and Extraction of Plant Materials

Plant materials were harvested in Cameroon and identified as *Alchornea latifolia*, *Annona senegalensis*, *Cananga odorata*, *Senna alata, Drypetes principum*, *Ficus benjamina*, *Ficus exasperata*, *Occimum gratissimum*, *Terminalia catappa*, and *Terminalia mantaly* through the National Herbarium of Cameroon, Yaoundé, where voucher specimens have been deposited under specific reference numbers. Plant names and specimen numbers, site and year of collection, traditional uses, parts used, extract codes and yields are summarized in Table 1. 

The plant samples were air-dried and ground into fine powder using an electric mill (Hammer Mill, Leabon 9FQ, Zhengzhou, China). One hundred grams of powder of each plant part were separately macerated in 1 L of (1) distilled water, (2) ethanol, (3) ethanol-water (70/30), (4) ethyl acetate, and (5) methanol, respectively, for 72 h at room temperature (27–29 °C). The organic macerates were filtered and filtrates evaporated using a rotary evaporator (Rotavapor, BUCHI 071, Flawil, Switzerland) at 40 °C. Decoctions were prepared by boiling plant powders in the same proportion in distilled water (100 g/1 L) for 2 h. Cold-extracted and decocted aqueous extracts were lyophilized at the Laboratory of Phytochemistry, Institute for Medical Research and Medicinal Plants Studies (IMPM), Yaoundé, Cameroon using a Virtis Wizard 2.0 Freeze Dryer Lyophilizer: Model: XLS-70 (Usitfroid, Maurepas, France). The extracts obtained were stored at 4 °C until bioassay.

### 2.2. Plasmodium Falciparum Culture and Maintenance

*P. falciparum* (chloroquine-sensitive 3D7 (*Pf*3D7) and resistant INDO (*Pf*INDO) strains) were maintained in culture using the method of Trager and Jensen [32] with some modifications [25]. Parasites were cultured in fresh O^+ve^ human erythrocytes suspended at 4% (*v*/*v*) hematocrit in complete RPMI 1640 medium (16.20 g/L RPMI 1640 (Sigma, Munich, Germany) containing 25 mM HEPES, 11.11 mM glucose, 0.20% sodium bicarbonate (Sigma, Munich, Germany), 0.50% Albumax I (Gibco, Waltham, MA, USA), 45 μg/mL hypoxanthine (Sigma, Munich, Germany) and 50 μg/mL gentamicin (Gibco, Waltham, MA, USA) and incubated at 37 °C in an atmosphere of 5% O_2_, 5% CO_2_, and 90% N_2_. The spent medium was replaced with fresh complete medium every day to propagate the culture. Giemsa-stained blood smears were examined microscopically under oil immersion to monitor cell-cycle transition and parasitemia.

### 2.3. In Vitro Anti-Plasmodial Assay

Plant extracts were assessed for in vitro antiplasmodial activity using the SYBR green I-based fluorescence assay set up as described by Smilkstein et al. [33]. Crude extracts were prepared at 25 mg/mL in dimethyl sulfoxide (DMSO), while the chloroquine (Sigma-Aldrich, New Delhi, India) stock solution used as standard drug was prepared in water (Milli-Q grade) at 1 mM. All stock solutions were then diluted in 96-well, round-bottom, tissue culture-grade plates (Corning, New York, USA) with fresh RPMI 1640 culture medium to achieve the required concentrations for testing. In all cases, except for chloroquine (positive control), the final solution contained 0.4 % DMSO, which was found to be non-toxic to the parasite. Extracts were tested at concentrations ranging from 0.10 to 100 μg/mL, and chloroquine was used at 1 µM. All tests were performed in triplicate. 

Briefly, 100 μL of sorbitol-synchronized parasites [34] were incubated under normal culture conditions (37 °C, 5% CO_2_, 5% O_2_, 90% N_2_) at 1% parasitemia and 2% hematocrit in flat-bottomed, 96-well plates (Corning, Corning, NY, USA) in the absence or presence of increasing concentrations of crude extracts for 48 h. Chloroquine (Sigma-Aldrich, New Delhi, India) was used as positive control, while 0.4% (*v*/*v*) DMSO was used as the negative control. Following incubation, 100 μL of SYBR green I lysis buffer (Tris (20 mM, pH 7.5), EDTA (5 mM), saponin (0.008%, *w*/*v*), and Triton X-100 (0.08%, *v*/*v*)) was added to each well and mixed gently twice, and incubated in dark at 37 °C for 1 h. Fluorescence was then measured with a Victor fluorescence multi-well plate reader (Perkin Elmer, Waltham, MA, USA) with excitation and emission wavelength bands centered at 485 and 530 nm, respectively. The fluorescence counts were plotted against drug concentration and the 50% inhibitory concentration (IC_50_) was determined by analysis of dose–response curves using the IC Estimator-version 1.2 software (http://www.antimalarial-icestimator.net/MethodIntro.htm) (Free Software Foundation, Boston, MA, USA). Resistance indices (RIs) were calculated as IC_50_*Pf*INDO/IC_50_*Pf*3D7. Results were validated microscopically by examination of Giemsa-stained smears of extract-treated/untreated parasite cultures. 

### 2.4. Cytotoxicity Study of the Selected Extracts Using MTT Assay

The cytotoxic effect of antiplasmodial extracts was assessed using the MTT assay [35], targeting human embryonic kidney cells (HEK239T cells) cultured in complete medium containing 13.5 g/L DMEM (Gibco, Waltham, MA USA), 10% fetal bovine serum (Gibco, Waltham, MA USA), 0.21% sodium bicarbonate (Sigma-Aldrich, New Delhi, India) and 50 μg/mL gentamicin (Gibco, Waltham, MA, USA). Essentially, HEK239T cells at 104 cells/200 μL/well were seeded into 96-well flat-bottomed tissue culture plates (Corning, Corning, NY, USA) in complete medium. Then, 50 µL of serially diluted extracts solutions (≤200 µg/mL) were added after 24 h of seeding and the samples incubated for 48 h in a humidified atmosphere at 37 °C and 5% CO_2_. DMSO at final concentrations (*v*/*v*) of 0.4% and 10% were used as negative (100% growth) and positive (0% growth) controls respectively. Twenty microliters of a stock solution of MTT (5 mg/mL in 1× phosphate-buffered saline) were added to each well, gently mixed, and incubated for additional 4 h. After spinning the plate at 1500 rpm for 5 min, the supernatant was carefully removed and 100 μL of 10% DMSO (*v*/*v*) was added. Formazan formation was read on a microtiter plate reader (Versa Max Microplate Reader, Molecular Devices, San Jose, CA, USA) at 570 nm. The 50% cytotoxic concentrations (CC_50_) of extracts were determined by analysis of dose response curves (Graphpad prism 5.0, GraphPad, La Jolla, CA, USA). Selectivity indices (CC_50_/IC_50_) were calculated for each extract.

## 3. Results and Discussion

Medicinal plants have and will always play a vital role in the management of community health and the discovery of novel chemotherapeutic agents since they are rich repositories of a wide range of metabolites that have promise against diverse diseases. Therefore, collection of plants based on the ethnomedical knowledge is still an attractive starting point for drugs discovery. In the present study, 10 medicinal plants from Cameroon were evaluated for their antiplasmodial activity against chloroquine (CQ)-sensitive *P. falciparum* 3D7 and CQ-resistant *P. falciparum* INDO strains. 

**Figure 1:**
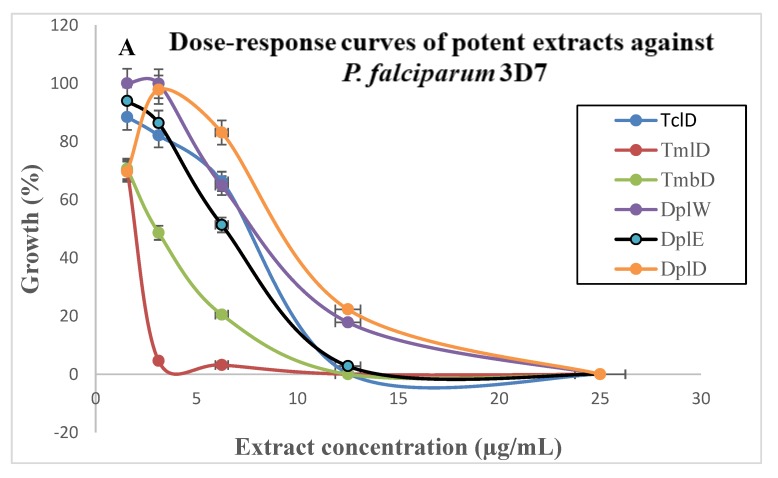

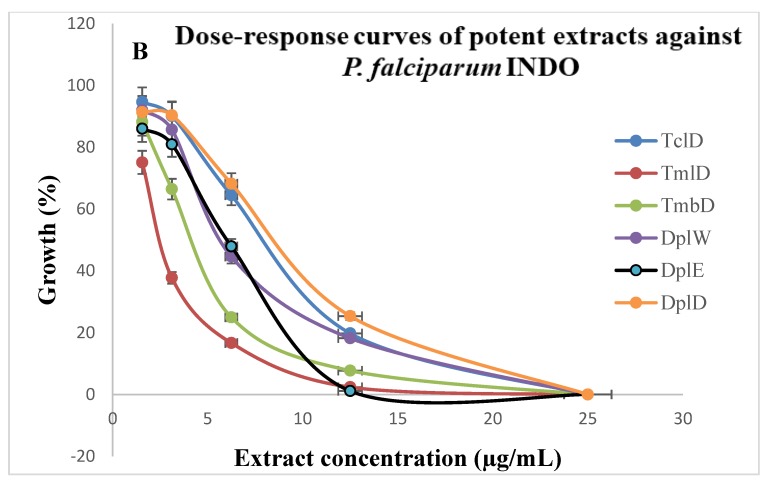
Dose–response curves of potent extracts from *Drypetes principum*, *Terminalia catappa*, and *Terminalia mantaly* on *Plasmodium falciparum* 3D7 (**A**) and INDO (**B**) strains. *Tc*l ^D^: Decoction of leaves of *T. catappa*; *Tm*l ^D^: Decoction of leaves of *T. mantaly*; *Tm*b ^D^: Decoction of bark of *T. mantaly*; *Dp*l ^W^: Aqueous maceration extract of leaves of *D. principum*; *Dp*l ^E^: Ethyl acetate extract of leaves of *D. principum*; *Dp*l ^D^: Decoction of leaves of *D. principum.*

The extraction yields as indicated in table 1 varied from 1.10% to 29.17%, depending on the plant part and solvent of extraction. The highest yields were obtained with the aqueous maceration extract of fruit of *Ficus benjamina* (*Fb*fr ^W^: 29.17%), followed by decoction extract of leaves of *T. mantaly* (*Tm*l ^D^: 27.70%) and aqueous maceration extract of leaves of *F. benjamina* (*Fb*l ^W^: 26.71%). The results of the in vitro evaluation of the potential of the 66 extracts from medicinal plants to inhibit the growth of the *Pf*3D7 and *Pf*INDO strains are presented in Figure 1 and summarized in Table 2. The antiplasmodial activity of plant extracts ranged from 1.90 to >100 μg/mL against the two strains. Bagavan et al. [36] have classified the antiplasmodial activity of plant extracts as good (IC_50_ < 10 μg/mL), moderate (IC_50_ > 10 to <25 μg/mL), and weak (IC_50_ > 25 μg/mL). Therefore, out of the 66 extracts tested, six showed good activity (IC_50_ = 2.49–6.49 μg/mL), 10 exhibited moderate activity (IC_50_ = 12.41–25.08 μg/mL), while 50 displayed weak (IC_50_ > 25 μg/mL) antiplasmodial activity against the malaria parasites (Figure 1A,B). All extracts tested were nearly equipotent against both sensitive and resistant strains of the malaria parasite.

The most active extracts were the water maceration and decoction, and ethyl acetate extracts of leaves of *D. principum* (IC_50_3D7/INDO = 4.91/6.64, 5.49/5.98, and 6.49/7.10 μg/mL respectively), water decoction extract of leaves of *T. catappa* (IC_50_3D7/INDO = 6.41/8.10 μg/mL) and water decoction extracts of leaves and bark of *T. mantaly* (IC_50_3D7/INDO = 2.49/1.90 and 3.70/2.80 μg/mL, respectively). As shown in Table 2, the promising extracts listed above exhibited no cytotoxicity on HEK293T at up to 200 μg/mL, giving selectivity indices (SI) in the range of >31.20–80.32. 

The antiplasmodial activity of *Drypetes principum* against both *Pf*3D7 and *Pf* INDO strains is being reported for the first time. For this plant and considering the criteria of Bagavan et al. [36], the water maceration (*Dp*st ^w^) and decoction (*Dp*st ^D^) extracts of stems showed no activity (IC_50_ > 100 µg/mL), contrasting with the water maceration and decoction extracts of the twig (*Dp*tw ^W^; *Dp*tw^D^) that exerted weak activity, the ethyl acetate extracts of twigs and stems (*Dp*tw ^E^; *Dp*st ^E^) that exhibited moderate activity and finally the promising extracts (water maceration of the leaf: *Dp*l^W^-IC_50_*Pf3D7*/INDO = 4.91/6.64 µg/mL; ethyl acetate extract of the leaf: *Dp*l^E^-IC_50_*Pf3D7*/INDO = 5.49/5.98 µg/mL; and the decoction of the leaf: *Dp*l^D^-IC_50_*Pf3D7*/INDO= 6.49/7.10 µg/mL) that showed good activity (IC_50_ < 10 µg/mL). Extracts from the leaves were promising given their potent activities in both polar water extracts and less polar ethyl acetate extract. Unlike leaves, the activities associated with extracts from twigs were solvent-dependent, ranging from moderate when using ethyl acetate to weak with aqueous extracts. The ethyl acetate extracts of twigs and stems were more active than their respective water extracts counterparts, suggesting that their antiplasmodial metabolites are more soluble in ethyl acetate than water. However, as stated earlier, independently of the solvent used, extracts from leaves exhibited good activities, indicating that the leaves of *D. principum* may contain various classes of antiplasmodial metabolites that are soluble in water (whether macerated or whether decocted) and in ethyl acetate, or the same metabolites may dissolve in both solvents due to their amphipathic nature.

Moreover, decoction extracts from the leaves of *T. catappa* and leaves and bark of *T. mantaly* exhibited very promising activity against *Pf* 3D7 (IC_50_ = 2.49–6.41 µg/mL) and *Pf*INDO (IC_50_ = 1.90–8.10 µg/mL). Earlier, Abiodun et al. [24] reported that hexane, ethyl acetate and methanol extracts of leaves of *T. catappa* showed potent antipasmodial activity against *Pf*K1(IC_50_ = 3.05–10.10 µg/mL) and *Pf*NF54 (IC_50_= 6.68–21.93 µg/mL) with hexane extract being less potent (IC_50_ = 10.10–21.93 µg/mL). A previous study by Mbouna et al. [27] showed that water and methanol extracts of leaves, stem bark, and roots of *T. mantaly* displayed very good activity against *Pf*3D7 (IC_50_ = 1.03–5.09 µg/mL) and *Pf*INDO (IC_50_ = 0.26–7.01 µg/mL). Equally, in vitro antiplasmodial activity of other species of the genus *Terminalia* has been previously reported [37,38]. Thus, extracts from stem bark of *Terminalia avicennoides* (IC_50_ = 10.99–14.76 µg/mL (*Pf*3D7) and 9.31–12.56 µg/mL (*Pf*K1) and isolated compounds including ellagic acid (IC_50_ = 12.14 and 11.20 µg/mL), flavogallonic acid (IC_50_ = 8.89 and 8.35 µg/mL), punicalagin (IC_50_ = 9.42 and 8.79µg/mL), castalagin (IC_50_ = 10.57 and 9.63 µg/mL) and terchebulin (IC_50_ = 8.89 and 8.49 µg/mL) have showed in vitro activity against *P. falciparum* 3D7 and K1, respectively [38].

## 4. Discussion

Ethyl acetate extracts of stems and twigs of *Alchornea lacifolia* (*Al*st ^E^, *Al*tw ^E^, respectively) displayed moderate antiplasmodial activity (IC_50_*Pf*3D7/INDO ranging 12.44–16.64 µg/mL) against both *P. falciparum* strains, whereas the corresponding aqueous extracts were weakly active or inactive (>25 to >100 μg/mL). Moreover, leaf and trunk extracts displayed weak antiplasmodial activity to inactivity against the sensitive and resistant *P. falciparum* strains. Okokon et al. [26] have recently reported that ethyl acetate extract of roots of *A. lacifolia* exhibits weak activity against both strains of *P. falciparum* with IC_50_ values of 38.44 μg/mL (*Pf* 3D7) and 40.17 μg/mL (*Pf* INDO) which suggests that stems and twigs may be preferred over roots and leaves of *A. lacifolia* as sources of antiplasmodial metabolites. 

Activity of extracts from *A. senegalensis* varied considerably depending of extract type and parasite strain, but none of them exhibited good antiplasmodial potency (IC_50_ < 10μg/mL). However, moderate activity was recorded for the ethanol and hydroethanol extracts of bark (*As*b ^Et^, *As*b ^Wet^) and hydroethanol extracts of stems and leaves (*As*st ^Wet^, *As*l ^Wet^) with IC_50_ ranging 13.16–25.08 μg/mL against both *P. falciparum* strains. Of note, the maceration extract of *A. senegalensis* bark (*As*b ^W^) showed moderate activity against the sensitive *Pf*3D7 strain but was rather inactive against the resistant INDO strain (IC_50_ > 100 μg/mL). Wele et al. [21] have recently reported moderate to weak antiplasmodial activity of ethanol extracts from leaves of *A. senegalensis* against *Pf*3D7 and *Pf*Dd2 (IC_50_ = 23.93 and 29.47 μg/mL respectively). However, leaf ethanolic crude extract of *A. senegalensis* growing in the Democratic Republic of Congo was reported to exhibit weak activity against *P. falciparum* FcM29 (IC_50_ = 32.52 µg/mL) [39], corroborating the findings of Ndjonka et al. [40] who reported weak antiplasmodial activity (IC_50_ = 94.80 µg/mL) of ethanol extract of leaves of *A. senegalensis* collected in Cameroon. In comparison to literature data, our findings suggest that hydro-ethanol may be the best solvent for extraction of antiplasmodial compounds from *A. senegalensis* leaves, twigs, and stems, while ethanol could be more appropriate for extraction of active compounds from the bark. 

Water and hydroethanol extracts of leaves, stems, and fruit as well as decoction extracts of *Ficus benjamina* were investigated against *P. falciparum* strains. Overall, the results showed that only the water maceration extract of leaves (*Fb*l ^W^) could exhibit moderate (IC_50_ =12.41 μg/mL) to weak (IC_50_ = 26.35 μg/mL) activity against *Pf*3D7 and *Pf*INDO, respectively. A weak activity was also recorded against *Pf*INDO for the hydroethanol extract of stems (*Fb*st ^Wet^) with IC_50_ of 52.91 μg/mL, but this extract showed to be inactive against the sensitive *P. falciparum* 3D7 strain (>100 μg/mL). In contrast to the present findings, Hayat et al. [22] reported that hydroethanol and petroleum ether leaf extracts of *F. benjamina* exhibited weak (IC_50_ = 31.80 µg/mL) and moderate (IC_50_ = 14.50 µg/mL) antiplasmodial effects against *Pf*3D7. Extracts of the other investigated species of *Ficus* genus (*F. exasperata*) exhibited mostly weak antiplasmodial activity (IC_50_ > 25 μg/mL) against both *P. falciparum* strains. 

The extracts of *Senna alata* were mostly inactive, with the exception of the leaf ethanol and decoction extracts (*Ca*l^Et^, *Ca*l^D^) that showed weak antiplasmodial activity against both strains (IC_50_ > 31.36 μg/mL) and the twig ethanol extract that also weakly inhibited the resistant *Pf*INDO (IC_50_ = 37.06 μg/mL) strain. Our findings corroborate those of Zirihi et al. [41] who recorded no antiplasmodial activity at concentrations up to 50 μg/mL in the leaf ethanol extract of *Senna alata.* Besides, Kayembe et al. [23] reported promising antiplasmodial activity (IC_50_ = 12.50 μg/mL) in the seed ethanol extract of *C. alata*. In addition, Kaushik et al. [25] reported antiplasmodial activity in the ethyl acetate extract of *C. alata* against both CQ-sensitive 3D7 (IC_50_ = 18.00 μg/mL) and CQ-resistant INDO (IC_50_ = 20.00 μg/mL) strains. This suggests that for this plant, ethyl acetate might be a better solvent for extraction of promising metabolites from the leaves as compared to ethanol and water.

This study also shows that the ethanol and methanol extracts of *Occimum* *gratissimum* have weak antiplasmodial activity (IC_50_ > 25 μg/mL) against both 3D7 and INDO strains. Abiodun et al. [19] also reported comparable activity profile (IC_50_ = 36.71 μg/mL) of methanol extract of *O.* *gratissimum* leaves against the chloroquine-sensitive *Pf*NF54 strain. The same authors further reported very good activity from ethyl acetate extract of leaves of *O. gratissimum* against *Pf*K1 (IC_50_ = 1.80 µg/mL) and *Pf*NF54 (IC_50_ = 3.61 µg/mL) respectively [24]. This activity variation indicates that instead of alcohols like ethanol and methanol, ethyl acetate should be the solvent of choice for extracting potent antipasmodial compounds from the leaves and roots of *O. gratissimum.* The results achieved also indicated that the decoction extract of *Cananga odorata* flower was inactive (IC_50_ ˃ 100 µg/mL) against both *Pf*3D7 and *Pf*INDO. Similar work undertaken by Nyugen-Poupin et al. [42], but targeting leaves instead, and using cyclohexane as extractant showed a moderate activity against *Pf*FcB1 strain with IC_50_ = 12.50 µg/mL. Overall, reported differences in the antiplasmodial activity of plant extracts may result from the influence of many factors such as time and site of plant collection, maturity of plant parts, intra-species variations, part investigated, edaphic substrate, climate, methods used for extraction, type of bioassay, parasite strain etc. [43].

The resistance index (RI) which indicates the inhibitory potential of a drug against both sensitive and resistant strains of *P. faciparum* was determined for each extract using IC_50_ values against *Pf*3D7 and *Pf*INDO strains. The RI ranged from 0.43 to ˃6.91. Given that extracts with RI ≤ 1 might be considered promising against both sensitive and resistant parasite strains, 16 extracts, including *Al*tw ^E^ (RI = 0.74), *As*l ^WEt^ (RI = 0.56), *As*l ^Et^ (RI = 0.72), *As*b ^Et^ (RI = 0.84), *As*l ^D^ (0.69), *As*b ^WEt^ (RI = 0.52), *Dp*st ^W^ (RI = 0.60), *Dp*tw ^D^(0.87), *Fe*st ^WEt^ (RI = 0.48), *Fe*st ^D^ (0.43), *Og*l ^Et^ (RI = 0.50), *Og*l ^M^ (RI = 0.45), *Og*r ^Et^ (RI = 0.55), *Og*r ^M^ (RI = 0.44), *Tm*l ^D^ (RI = 0.76), *Tm*b ^D^ (RI = 0.75) exhibited very good resistance indexes (overall RI < 0.90) and were categorized as of interest. This indicates that investigating each of these extracts in details might lead to identification of potent chemical entities as starting points for further drug discovery research for the ultimate goal of controlling both sensitive and resistant strains of *Plasmodium falciparum*. 

Selectivity index (SI), defined as the ratio of CC_50_ HEK293T to IC_50_ *P. falciparum* was also determined. The higher the SI, the more promising is the extract due to its selective action on malaria parasites. Eleven out of the 14 selected plant extracts that were evaluated for cytotoxicity displayed strong selectivity (SI > 10.58) for *P. falciparum*. The highest SI values were obtained for the decoction extract of leaves and bark of *T. mantaly* (SI ˃ 80.32). Overall, water, ethyl acetate, and decoction extracts of leaves of *D. principum*, and decoction extracts of leaves of *T. catappa* and *T. mantaly* are considered of interest since they display high antiplasmodial activity (IC_50_ = 1.90–8.10 µg/mL) with high selectivity indices (SI ˃ 31.20) against both *P. falciparum* 3D7 and INDO strains. 

## 5. Conclusions

The need to continue searching for new antimalarial molecules is driven by the continuous spread of multi-drug resistant malaria parasites. The present study has found that the leaves of *D. principum*, and *T*. *catappa* and bark of *T*. *mantaly* possess significant antiplasmodial activities, with good selectivity against chloroquine-sensitive and -resistant strains of *P. falciparum*. These findings confirm the use of much of these plants in the treatment of malaria and related symptoms. Further studies on these extracts, including bioassay-guided fractionation, are likely to yield new antimalarial compounds and ancillary molecules which could be developed as alternative drug combination therapies against malaria.

## Figures and Tables

**Table 1:** Information on plant species and extraction yields.

	Names of Plant Species (Family) and Voucher Specimen Number	Local Name	Collection Site in CAMEROON (Year)	Main Traditional Uses	Part Used	Extract Code	Extraction Yield (% *w*/*w*) ^#^
1	*Alchornea lacifolia* (Euphorbiaceae) 601610/HNC	Eboe	Mount Kalla (2014)	Malaria, inflammation, and infectious diseases [16,28]	Leaf	*Al*l ^E^	5.90
						*Al*l ^D^	4.50
					Twig	*Al*tw ^W^	9.70
						*Al*tw ^E^	5.43
						*Al*tw ^D^	6.20
					Stem	*Al*st ^W^	8.31
						*Al*st ^E^	6.81
						*Al*st ^D^	7.91
					Trunk	*Al*tr ^W^	7.98
						*Al*tr ^D^	8.95
2	*Annona senegalensis* (Annonaceae) 32071/HNC	African custard apple	Bafia (2015)	Fever, diarrhea, joints and respiratory diseases, conjunctivitis, wounds, trypanosomiasis, jaundice, hemorrhoids, convulsions, ovarian cancer, and asthenia [20]	Bark	*As*b ^W^	6.80
						*As*b ^Et^	7.00
						*As*b ^WEt^	8.20
						*As*b ^D^	7.82
					Leaf	*As*l ^WEt^	8.70
						*As*l ^W^	10.00
						*As*l ^Et^	16.70
						*As*l ^D^	15.59
					Twig	*As*tw ^WEt^	6.10
						*As*tw ^Et^	9.20
						*As*tw ^W^	4.30
						*As*tw ^D^	3.40
					Stem	*As*st ^WEt^	2.00
						*As*st ^Et^	4.70
						*As*st ^W^	1.10
						*As*st ^D^	2.12
3	*Cananga odorata* (Annonaceae) 42250/HNC	Ylang ylang	Yaoundé (2014)	Fever, malaria, hepatitis, anxiety, itches, tension, shock, fear and panic [17]	Flower	*Co*fl ^D^	20.02
4	*Drypetes principum* (Euphorbiaceae) 52007/HNC	ND	Mount Kalla (2014)	ND	Leaf	*Dp*l ^W^	12.19
						*Dp*l ^D^	19.28
						*Dp*l ^E^	10.21
					Twig	*Dp*tw ^W^	9.76
						*Dp*tw ^E^	8.90
						*Dp*tw ^D^	7.47
					Stem	*Dp*st ^W^	7.65
						*Dp*st ^D^	8.43
						*Dp*st ^E^	6.56
5	*Ficus benjamina* (Moraceae) 65599/HNC	ND	Yaoundé (2015)	Malaria and other parasitic diseases [22]	Fruit	*Fb*fr ^WEt^	19.37
						*Fb*fr ^W^	29.17
						*Fb*fr ^D^	23.21
					Leaf	*Fb*l ^WEt^	21.45
						*Fb*l ^W^	26.71
						*Fb*l ^D^	24.54
					Stem	*Fb*st ^WEt^	10.18
						*Fb*st ^W^	7.33
						*Fb*st ^D^	6.45
6	*Ficus exasperata* (Moraceae) 19095/HNC (YA)	Lewoua	Yaoundé (2015)	Malaria, dysentery, hemorrhoids, and urinary infections [18]	Leaf	*Fe*l ^W^	19.23
						*Fe*l ^WEt^	20.45
						*Fe*l ^D^	19.56
					Stem	*Fe*st ^W^	8.10
						*Fe*st ^WEt^	11.89
						*Fe*st ^D^	9.36
7	*Occimum gratissimum* (Lamiaceae) 5817/SRF/Cam	Messep	Yaoundé (2015)	Headaches, giddiness, cold and cough, headache, fever, ophthalmic, skin diseases, and pneumonia, diarrhea, dysentery, piles, and convulsions [29,30]	Leaf	*Og*l ^Et^	8.87
						*Og*l ^M^	10.21
					Root	*Og*r ^Et^	9.11
						*Og*r ^M^	8.32
					Stem	*Og*st ^Et^	7.69
						*Og*st ^M^	10.80
8	*Senna alata* (Fabaceae) 1871/HNC (YA)	Ngom-Ntam Ndawolo	Yaoundé (2015)	Yellow fever, malaria, diabetes, constipation, hemorrhoids, inguinal hernia, blennorrhagia, and syphilis [31]	Leaf	*Ca*l ^Et^	12.10
						*Ca*l ^D^	10.34
					Stem	*Ca*st ^W^	9.20
						*Ca*st ^D^	7.90
					Twig	*Ca*tw ^Et^	7.50
						*Ca*tw ^W^	10.01
						*Ca*tw ^D^	9.15
9	*Terminalia catappa* (Combretaceae) 51244/HNC	Tropical almond	Yaoundé (2015)	Fever, diaphoretic, amoebiasis, mouth infections, leprosy, headaches, wounds, gonorrhea and anemia [14,15]	Leaf	*Tc*l ^D^	20.27
10	*Terminalia mantaly* (Combretaceae) 64212/HNC	-	Yaoundé (2015)	Gastroenteritis, hypertension, diabetes, oral and skin conditions, oral and genital candidiasis [17,27]	Leaf	*Tm*l ^D^	27.70
					Bark	*Tm*b ^D^	23.10

^#^ The % yield (*w*/*w*) of extraction was calculated from the weight of extract relative to 100 g of starting plant material. *Annona senegalensis* (*As*) (*As*b ^WEt^: Hydroethanol extract of bark of *As*; *As*b ^W^: Aqueous maceration extract of bark of *As*; *As*b ^D^: Decoction extract of bark of *As*; *As*b ^Et^: Ethanol extract of bark of *As*; *As*tw ^WEt^: Hydroethanol extract of twigs of *As*; *As*tw ^W^: Aqueous maceration extract of twigs of *As*; *As*tw ^D^: Decoction extract of twigs of *As*; *As*tw ^Et^: Ethanol extract of twigs of *As*; *As*st ^WEt^: Hydroethanol extract of stems of *As*; *As*st ^W^: Aqueous maceration extract of stems of *As*; *As*st ^D^: Decoction extract of stems of *As*; *As*st ^Et^: Ethanol extract of stems of *As*; *As*l ^WEt^: Hydroethanol extract of leaves of *As*; *As*l ^W^: Aqueous maceration extract of leaves of *As*; *As*l ^Et^: Ethanol extract of leaves of *As*; *As*l ^D^: Decoction extract of leaves of *As*). *Alchornea latifolia* (*Al*) (*Al*st ^E^: Ethyl acetate extract of stems of *Al*; *Al*st ^D^: Decoction extract of stems of *Al*; *Al*st ^W^: Aqueous maceration extract of stems of *Al*; *Al*tw ^W^: Aqueous maceration extract of twigs of *Al*; *Al*tw ^D^: Decoction extract of twigs of *Al*; *Al*tw ^E^: Ethyl acetate extract of twigs of *Al*; *Al*tr ^W^: Aqueous maceration extract of the trunk of *Al*; *Al*tr ^D^: Decoction extract of the trunk of *Al*; *Al*l ^D^: Decoction extract of leaves of *Al*; *Al*l ^E^: Ethyl acetate extract of leaves of *Al*). *Cananga odorata* (*Co*) (*Co*fl ^D^: Decoction extract of flowers of *Co*). *Senna alata* (*Ca*) (*Ca*tw ^Et^: Ethanol extract of twigs of *Ca*; *Ca*tw ^W^: Aqueous maceration extract of twigs of *Ca*; *Ca*tw ^D^: Decoction extract of twigs of *Ca*; *Ca*l ^Et^: ethanol extract of leaves of *Ca*; *Ca*l ^D^: Decoction extract of leaves of *Ca*; *Ca*st ^D^: Decoction extracts of stems of *Ca*; *Ca*st ^W^: Aqueous maceration extracts of stems of *Ca*). *Drypetes principum* (*Dp*) (*Dp*tw ^E^: Ethyl acetate extract of twigs of *Dp*; *Dp*tw ^W^: Aqueous maceration extract of twigs of *Dp*; *Dp*tw ^D^: Decoction extract of twigs of *Dp*; *Dp*l ^W^: Aqueous maceration extract of leaves of *Dp*; *Dp*l ^E^: Ethyl acetate extract of leaves of *Dp*; *Dp*l ^D^: Decoction extract of leaves of *Dp*; *Dp*st ^D^: Decoction extract of leaves of *Dp*; *Dp*st ^E^: Ethyl acetate extract of stems of *Dp*; *Dp*st ^D^: Decoction extract of stems of *Dp*). *Ficus benjamina* (*Fb*) (*Fb*fr ^WEt^: Hydroethanol extract of fruit of *Fb*; *Fb*fr ^W^: Aqueous maceration extract of fruit of *Fb*; *Fb*fr ^D^: Decoction extract of fruit of *Fb*; *Fb*l ^W^: Aqueous maceration extract of leaves of *Fb*; *Fb*l ^D^: Decoction extract of leaves of *Fb*; *Fb*st ^W^: Aqueous maceration extract of stems of *Fb*; *Fb*st ^D^: Decoction extract of stems of *Fb*; *Fb*st ^WEt^: Hydroethanol extract of stems of *Fb*). *Ficus* *exasperata* (*Fe*) (*Fe*l ^W^: Aqueous maceration extract of leaves of *Fe*; *Fe*l ^D^: Decoction extract of leaves of *Fe*; *Fe*l ^Wet^: Hydroethanol extract of leaves of *Fe*; *Fe*st ^WEt^: Hydroethanol extract of *Fe*; *Fe*st ^D^: Decoction extract of stems of *Fe*; *Fe*st ^W^: Aqueous maceration extract of stems of *Fe*). *Occimum gratissimum* (*Og*) (*Og*st ^Et^: Ethanol extract of stems of *Og*; *Og*st ^M^: Methanol extract of stems of *Og*; *Og*l ^Et^: Ethanol extract of leaves of *Og*; *Og*l ^M^: Methanol extract of leaves of *Og*; *Og*r ^M^: Methanol extract of roots of *Og*; *Og*r ^Et^: Ethanol extract of roots of *Og*). *Terminalia catappa* (*Tc*) (*Tc*l ^D^: Decoction extract of leaves of *Tc*). *Terminalia mantaly* (*Tm*) (*Tm*l ^D^: Decoction extract of leaves of *Tm*; *Tm*b ^D^: Decoction extract of bark of *Tm*).

**Table 2:** Antiplasmodial activity and cytotoxicity of plant extracts.

Plant Species (Family)	Extracts	*P. falciparum* (IC_50_ µg/mL)			^4^ CC_50_ (µg/mL)	^5^ SI (CC_50_/IC_50_)	
		^1^*Pf*3D7	^2^*Pf*INDO	^3^ RI		*Pf*3D7	*Pf*INDO
***Alchornea Lacifolia* (Euphorbiaceae) 601610/HNC**	*Al*st ^E^	14.88 ± 0.12	15.64 ± 0.63	1.05	>200	>13.44	>12.78
	*Al*st ^W^	>100	>100	-	-	-	-
	*Al*st ^D^	>100	>100	-	-	-	-
	*Al*tw ^W^	38.42 ± 0.46	40.20 ± 1.61	1.04	-	-	-
	*Al*tw ^E^	16.64 ± 0.63	12.44 ± 0.33	0.74	>200	>12.01	>16.33
	*Al*tw ^D^	48.42 ± 0.60	54.20 ± 0.61	1.11	-	-	-
	*Al*tr ^W^	>100	>100	-	-	-	-
	*Al*tr ^D^	>100	>100	-	-	-	-
	*Al*l ^E^	41.38 ± 0.36	50.83 ± 1.60	1.22	-	-	-
	*Al*l ^D^	49.80 ± 0.45	56.83 ± 1.01	1.14	-	-	-
***Annona Senegalensis* (Annonaceae) 32071/HNC**	*As*l ^Wet^	25.08 ± 0.30	14.09 ± 0.88	0.56	81.61 ± 0.48	3.25	5.79
	*As*l ^W^	>100	>100	−	-	-	-
	*As*l ^Et^	39.40 ± 0.80	28.72 ± 2.32	0.72	-	-	-
	*As*l ^D^	42.10 ± 0.90	29.20 ± 1.30	0.69	-	-	-
	*As*b ^W^	14.47 ± 0.30	>100	>6.91	-	-	-
	*As*b ^Et^	19.82 ± 1.82	16.80 ± 0.17	0.84	97.95 ± 0.25	4.94	5.82
	*As*b ^Wet^	25.07 ± 1.36	13.16 ± 0.00	0.52	>200	>7.97	>15.19
	*As*b ^D^	29.07 ± 1.60	30.60 ± 1.09	1.05	-	-	-
	*As*st ^W^	>100	>100	-	-	-	-
	*As*st ^Wet^	18.89 ± 0.46	20.20 ± 0.98	1.06	>200	>10.58	>9.90
	*As*st ^Et^	>100	>100	-	-	-	-
	*As*st ^D^	>100	>100	-	-	-	-
	*As*tw ^Wet^	30.41 ± 0.52	13.17 ± 0.00	0.43	-	-	-
	*As*tw ^Et^	>100	>100	-	-	-	-
	*As*tw ^W^	>100	>100	-	-	-	-
	*As*tw ^D^	>100	>100	-	-	-	-
***Cananga Odorata* (Annonaceae) 42250/HNC**	*Co*fl ^D^	>100	>100	-	-	-	-
***Drypetes Principum* (Euphorbiaceae) 52007/HNC**	*Dp*tw ^W^	31.52 ± 0.39	35.94 ± 2.75	1.14	-	-	-
	*Dp*tw ^E^	12.68 ± 0.00	12.74 ± 0.00	1.00	98.14 ± 0.48	7.73	7.70
	*Dp*tw ^D^	30.21 ± 0.91	26.40 ± 1.75	0.87	-	-	-
	*Dp*l ^W^	4.91 ± 0.29	6.64 ± 0.00	1.35	>200	>40.73	>30.12
	*Dp*l ^E^	5.49 ± 0.63	5.98 ± 0.40	1.08	>200	>36.43	>33.44
	*Dp*l ^D^	6.49 ± 0.58	7.10 ± 0.82	1.09	>200	>30.81	>28.16
	*Dp*st ^W^	>100	>100	-	-	-	-
	*Dp*st ^E^	27.78 ± 0.32	16.71 ± 0.25	0.60	-	-	-
	*Dp*st ^D^	>100	>100	-	-	-	-
***Ficus Benjamina* (Moraceae) 65599/HNC**	*Fb*fr ^Wet^	>100	>100	-	-	-	-
	*Fb*fr ^W^	>100	>100	-	-	-	-
	*Fb*fr ^D^	>100	>100	-	-	-	-
	*Fb*l ^W^	12.41 ± 0.36	26.35 ± 1.58	2.12	>200	>16.11	>7.59
	*Fb*l ^Wet^	>100	>100	-	-	-	-
	*Fb*l ^D^	>100	>100	-	-	-	-
	*Fb*st ^W^	>100	>100	-	-	-	-
	*Fb*st ^Wet^	>100	52.91 ± 2.29	NA	-	-	-
	*Fb*st ^D^	>100	>100	-	-	-	-
***Ficus Exasperate* (Moraceae) 19095/HNC (YA)**	*Fe*st ^W^	>100	>100	-	-	-	-
	*Fe*st ^Wet^	55.70 ± 0.50	27.22 ± 1.29	0.48	-	-	-
	*Fe*st ^D^	57.60 ± 0.40	25.12 ± 1.90	0.43	-	-	-
	*Fe*l ^W^	23.84 ± 0.48	28.00 ± 1.67	1.17	-	-	-
	*Fe*l ^Wet^	26.99 ± 0.60	35.41 ± 3.23	1.31	-	-	-
	*Fe*l ^D^	27.29 ± 0.60	39.41 ± 1.30	1.44	-	-	-
***Occimum Gratissimum*** **(Lamiaceae) 5817/SRF/Cam**	*Og*st ^Et^	>100	>100	-	-	-	-
	*Og*st ^M^	>100	46.36 ± 3.38	NA	-	-	-
	*Og*l ^Et^	54.41 ± 0.03	27.50 ± 2.56	0.50	-	-	-
	*Og*l ^M^	48.11 ± 0.68	21.79 ± 2.49	0.45	-	-	-
	*Og*r ^Et^	52.41 ± 1.20	29.01 ± 2.90	0.55	-	-	-
	*Og*r ^M^	54.22 ± 0.75	24.33 ± 1.13	0.44	-	-	-
***Senna Alata*** **(Fabaceae) 1871/HNC (YA)**	*Ca*tw ^Et^	>100	37.06 ± 1.80	NA	-	-	-
	*Ca*tw ^W^	>100	>100	-	-	-	-
	*Ca*tw ^D^	>100	>100	-	-	-	-
	*Ca*l ^Et^	31.36 ± 0.73	32.38 ± 2.84	1.03	-	-	-
	*Ca*l ^D^	41.60 ± 0.34	52.80 ± 1.40	1.26	-	-	-
	*Ca*st ^W^	>100	>100	-	-	-	-
	*Ca*st ^D^	>100	>100	-	-	-	-
***Terminalia Catappa* (Combretaceae) 51244/HNC**	*Tc*l ^D^	6.41 ± 0.43	8.10 ± 0.30	1.26	>200	>31.20	>24.69
***Terminalia Mantaly* (Combretaceae) 64212/HNC**	*Tm*l ^D^	2.49 ± 0.09	1.90 ± 0.10	0.76	>200	>80.32	>105.26
	*Tm*b ^D^	3.70 ± 0.16	2.80 ± 0.60	0.75	>200	>54.05	>71.42
**Chloroquine (µM)**		40	400	10	-	-	-

The extracts were screened against *P. falciparum*: ^1^ CQ-sensitive *Pf*3D7 and ^2^ CQ-resistant *Pf* INDO strains in culture and the activity expressed as IC_50_ from sigmoidal dose–response curves; SD: standard deviation, all data are mean values ± standard deviation from triplicate experiments; ^3^ Resistance index was calculated as the ratio of IC_50_-resistant strain to IC_50_-sensitive strain; ^4^ Cell cytotoxicity was evaluated against HEK239T mammalian cells; ^5^ SI: selectivity indices were calculated for each parasite strain; (-): not tested; NA: not applicable; D: decoction; E: ethyl acetate; Et: ethanol; M: methanol; W: water; WEt: hydroethanol; IC_50_: 50% inhibitory concentration.
